# *Methanothermobacter thermautotrophicus* modulates its membrane lipids in response to hydrogen and nutrient availability

**DOI:** 10.3389/fmicb.2015.00005

**Published:** 2015-01-22

**Authors:** Marcos Y. Yoshinaga, Emma J. Gagen, Lars Wörmer, Nadine K. Broda, Travis B. Meador, Jenny Wendt, Michael Thomm, Kai-Uwe Hinrichs

**Affiliations:** ^1^Organic Geochemistry Group, MARUM Center for Marine Environmental Sciences, University of BremenBremen, Germany; ^2^Department of Microbiology and Archaea Center, University of RegensburgRegensburg, Germany

**Keywords:** archaea, stress response, polar lipids, diether, tetraether

## Abstract

*Methanothermobacter thermautotrophicus* strain ΔH is a model hydrogenotrophic methanogen, for which extensive biochemical information, including the complete genome sequence, is available. Nevertheless, at the cell membrane lipid level, little is known about the responses of this archaeon to environmental stimuli. In this study, the lipid composition of *M. thermautotrophicus* was characterized to verify how this archaeon modulates its cell membrane components during growth phases and in response to hydrogen depletion and nutrient limitation (potassium and phosphate). As opposed to the higher abundance of phospholipids in the stationary phase of control experiments, cell membranes under nutrient, and energy stress were dominated by glycolipids that likely provided a more effective barrier against ion leakage. We also identified particular lipid regulatory mechanisms in *M. thermautotrophicus*, which included the accumulation of polyprenols under hydrogen-limited conditions and an increased content of sodiated adducts of lipids in nutrient-limited cells. These findings suggest that *M. thermautotrophicus* intensely modulates its cell membrane lipid composition to cope with energy and nutrient availability in dynamic environments.

## INTRODUCTION

Analysis of intact polar lipids (IPL) – membrane-associated lipids such as phospholipids and glycolipids – is one strategy for investigating microbial populations in culture and natural systems (e.g., Sturt et al., 2004), and is being presently applied to understand archaea in natural ecosystems (e.g., Biddle et al., 2006; Lipp et al., 2008; Rossel et al., 2011; Schubotz et al., 2011, 2013). Although the analysis of individual compounds by NMR is mandatory for strict structural identification, advances in IPL detection by high-performance liquid chromatography coupled to mass spectrometry (HPLC-MS) have revealed an unprecedented variety of archaeal lipids (Yoshinaga et al., 2011; Liu et al., 2012; Becker et al., 2013; Wörmer et al., 2013; Zhu et al., 2013, 2014). This structural diversity of IPL might reflect both the staggering diversity of archaea in nature and mechanisms of archaeal cell membrane adaptation in response to environmentally relevant conditions (e.g., energy and nutrient limitation).

It is well known that bacteria modify their membrane IPL composition in response to growth and environmental conditions (e.g., Zhang and Rock, 2008; Zavaleta-Pastor et al., 2010), however, there is little information on how and whether such adaptation occurs in archaea. To date, controlled experiments to observe changes in archaeal lipids or test hypotheses about membrane adaptations in archaea have only been reported for the effect of temperature, growth stage and pH (inferred from optimum pH of a range of organisms) for a handful of archaea (e.g., Kramer and Sauer, 1991; Uda et al., 2004; Lai et al., 2008; Matsuno et al., 2009; Boyd et al., 2011). For instance, the number of cyclopentane rings in tetraether moieties of thermophilic archaea varies with growth temperature, reflecting an adaptation to adjust their membrane fluidity (Uda et al., 2001; Gliozzi et al., 2002).

Even fewer studies have investigated these changes at the IPL level (Nicolaus et al., 1989; Morii and Koga, 1993; Uda et al., 2001; Shimada et al., 2008; Elling et al., 2014; Meador et al., 2014). Increasing amounts of glycolipids relative to phospholipids in *Thermoplasma acidophilum* were demonstrated to decrease proton permeability (Shimada et al., 2008). More recently, cells of *Thermococcus kodakarensis* were shown to accumulate intracellular phosphorous in IPL, when subject to phosphate-limited conditions (Meador et al., 2014). Here, we sought to expand on these findings using controlled conditions and *M. thermautotrophicus* as a model organism. *M. thermautotrophicus* is a hydrogenotrophic methanogen from anaerobic environments and represents perhaps the most studied archaeon among the diverse groups of methanogens.

Previous investigations of *M. thermautotrophicus* focused on lipid changes during growth stages have led to contradictory results. For example a decrease (Kramer and Sauer, 1991) or an increase (Morii and Koga, 1993) in the relative abundance of tetraether lipids has been observed for cells transitioning from exponential growth to stationary phase. In contrast to these studies, which quantified the lipid classes after chemical hydrolysis, here we performed a direct quantification of IPL during growth stages by HPLC-MS (Wörmer et al., 2013). Moreover, we examined whether *M. thermautotrophicus* adapts its membrane lipid composition in response to: (1) energy limitation (H_2_) and (2) nutrient limitation (potassium and phosphate). Hydrogen and nutrient availability have been shown to significantly impact the cell wall and membrane components of* M. thermautotrophicus* both at the gene expression (e.g., Kato et al., 2008) and the molecular levels [e.g., cyclic 2,3-diphosphoglycerate (cDPG), Krueger et al., 1986; cell wall thickness, Nakamura et al., 2006]. This study represents the first attempt to investigate how* M. thermautotrophicus* modulates its IPL composition in response to environmentally relevant conditions that affect its growth.

## MATERIALS AND METHODS

### CULTIVATION CONDITIONS AND SAMPLING

*Methanothermobacter thermautotrophicus* ΔH (DSM 1053) was grown at 65°C in 65 L bioreactors containing 50 L liquid medium. Standard medium was prepared anaerobically and contained per liter: 0.45 g NaCl, 5 g NaHCO_3_, 0.1 g MgSO_4_.7H_2_O, 0.225 g KH_2_PO_4_, 0.3 g K_2_HPO_4_.3H_2_O, 0.225 g (NH_4_)_2_SO4, 0.06 g CaCl_2_.2H_2_O, 0.002 g (NH_4_)_2_Ni(SO_4_)_2_, 0.002 g FeSO_4_.7H_2_O, 1 mg resazurin, final concentrations of 1x Wolfe’s vitamins and 1x Wolfe’s minerals, modified as outlined by Gagen et al. (2013) and 0.3 g Na_2_S.3H_2_O and 1.5 g cysteine hydrochloride monohydrate, as reducing agents. The pH was set to 7.5 using H_2_SO_4_. In reactors, 2 bar H_2_:CO_2_ (80:20 vol:vol) was provided as the energy and carbon source and gassing at approximately 1.5 L min^-1^ was started when the culture was between early- and mid-exponential phase. Further reducing agents were added to reactors and the gassing rate increased to approximately 2.5 L min^-1^ when the culture reached late exponential phase. Under these growth conditions, maximum cell concentrations of 5 × 10^8^ cells ml^-1^ were routinely achieved. Samples (2 L) were harvested from reactors at late-exponential phase (L-Exp), 3 h after reaching maximum cell concentration (early stationary phase, E-Stat) and then nine (mid, M-Stat) and 18 h later (late, L-Stat). Samples were harvested by centrifugation at 13,000 *g*, 30 min., 4°C. After cooling, the remaining 42 L of culture in the reactors were also harvested by continuous flow-through centrifugation (end harvest samples).

For hydrogen limitation experiments, the gas phase of the reactor was switched to N_2_:CO_2_ (80:20) when the maximal cell concentration was almost reached (i.e., 3 h before collection of sample E-Stat) and samples were collected at the same time points as for standard conditions (L-Exp to L-Stat). As such, samples L-Exp did not experience hydrogen-limited conditions, and they should thus be comparable to the control samples at this growth phase. In the nutrient limitation experiments, 2 bar H_2_:CO_2_ (80:20 vol:vol) was provided to *M. thermautotrophicus* that was adapted to and grown on medium with only 20 μM potassium and 10 μM phosphate available (substantially reduced compared to control medium, which offered ca. 4.3 and 3.0 mM, respectively). Samples from nutrient-limited experiments were only collected at L-Exp, E-Stat, and L-Stat. Gassing and addition of further reducing agents were not required for nutrient-limited cultures as neither altered the final maximal cell concentrations of only 2 × 10^7^ cells ml^-1^ achievable under these conditions. All experiments were performed in triplicate, and the different treatments are summarized in **Table 1**.

**Table 1:** Summary of experimental conditions (control, hydrogen- and nutrient-limited treatments) and respective cell concentrations (×10^8^ cells ml^-**1**^, in parenthesis the SD) of *Methanothermobacter thermautotrophicus* at: late exponential phase (L-Exp), early, mid, and late stationary phases (E-, M-, and L-Stat).

	Control				Hydrogen-limited				Nutrient-limited		
Headspace	80:20 (H_2_:CO_2_ vol:vol)				80:20 (H_2_:CO_2_) until L-Exp and				80:20 (H_2_:CO_2_ vol:vol)		
					80:20 (N_2_:CO_2_) 3 h prior to E-Stat						
Nutrients	4.3 and 3.0 mM				4.3 and 3.0 mM				20 and 10 μM		
	**L-Exp**	**E-Stat**	**M-Stat**	**L-Stat**	**L-Exp**	**E-Stat**	**M-Stat**	**L-Stat**	**L-Exp**	**E-Stat**	**L-Stat**
Cell	1.0	6.9	5.7	6.0	1.6	5.0	5.1	4.9	0.1	0.2	0.2
concentrations	(0.8)	(1.1)	(0.7)	(0.8)	(0.3)	(0.2)	(0.2)	(0.1)	(0.09)	(0.01)	(0.01)

*Headspace gas atmosphere (vol:vol) and concentrations of potassium and phosphate, respectively*.

### LIPID EXTRACTION AND ANALYSIS

Lipids were extracted according to Sturt et al. (2004) with slight modifications. In brief, samples were first lyophilized and weighed. Dry cell mass (0.02–0.25 g) was combined with pre-combusted sand (2 g) and extracted four times. Samples were extracted by ultrasonication into a solvent mixture (v:v) of methanol (MeOH), dichloromethane (DCM), and aqueous buffer (2:1:0.8). A phosphate buffer (8.7 g L^-1^ KH_2_PO_4_, pH 7.4) was used for the first two steps, and a trichloroacetic acid buffer (50 g L^-1^, pH 2) for the final two steps. Supernatants were pooled in a separation funnel and DCM and water were added in order to allow optimal phase separation. After transferring the organic phase, the aqueous phase was extracted three more times with DCM. Pooled organic layers were then washed three times with deionized milliQ water. The final extract was gently evaporated under N_2_ flow and stored at -20°C.

Chromatographic separation was achieved on a Waters Acquity BEH C_18_ column (Wörmer et al., 2013) with a Dionex Ultimate 3000RS UHPLC coupled to a maXis quadrupole time-of-flight mass spectrometer (Q ToF-MS, Bruker Daltonics, Bremen, Germany) equipped with an ESI source. Detection of lipids was performed in positive ionization mode while scanning a mass-to-charge (m/z) range from 150 to 2,000. MS^2^ scans were obtained in data-dependent mode, for each MS full scan up to three MS^2^ experiments, targeting the most abundant ions, were performed. Active exclusion limits the times a given ion is selected for fragmentation (three times every 0.5 min) and thus allowed to also obtain MS^2^ data of less abundant ions. Lipid identification was achieved by monitoring exact masses of possible parent ions (present as either H^+^, NH_4_^+^, or Na^+^ adducts) in combination with characteristic fragmentation patterns as outlined by Yoshinaga et al. (2011) and supported by compound identities revealed in previous studies (Kramer et al., 1987; Nishihara et al., 1987, 1989; Morii and Koga, 1994).

Lipid quantification was achieved by comparison of parent ion responses relative to known amounts of an internal standard (1,2-dihenarachidoyl-sn-glycero-3-phosphocholine, C_21_-PC, Avanti Lipids). Reported concentrations were corrected for response factors of commercially available standards (core archaeol, AR, and phosphoethanolamine archaeol, PE-AR, from Avanti Polar Lipids Inc., USA and phosphatidyl-glycerol-monoglycosyl glycerol-di-biphytanyl-glycerol-tetraether, PG-GDGT-G, from Matreya LLC, Pleasant Gap, PA, USA) and purified standards (core GDGT, G-GDGT, 2G-GDGT, G-AR, and 2G-AR) from *Archaeoglobus fulgidus* as described in Zhu et al. (2013). Among the tetraethers, all core GDGT and intact GDGT with more than one sugar and those with phosphate-bearing headgroups were quantified using the response factors of core GDGT, 2G-GDGT, and PG-GDGT-G, respectively. The abundance of intact AR with more than one sugar and phosphate-bearing headgroups were corrected, respectively, for the response factors of 2G-AR and PE-AR standards. The unmodified and glycosylated polyprenols (with additional modifications thereof) were corrected by their response factors relative to core AR and G-AR, respectively.

### CELLULAR CARBON

Aliquots of lyophilized cell pellets (0.5–2 mg) were subjected to hydrochloric acid (HCl) vapor overnight to remove inorganic carbon. Mass percentage of C was determined using a ThermoFinnigan Flash Elemental Analyzer 2000 (Bremen, Germany). In order to compare the distinct treatments, we applied the carbon to lipid ratio (e.g., Lipp et al., 2008). Although the cellular amounts of lipids and total carbon can vary widely, the ratio of cellular carbon and cell membrane lipids does not fluctuate widely with cell volume (ratio = 11–15, cf., Simon and Azam, 1989).

### STATISTICAL ANALYSES

The Simpson diversity index (*D*), typically used to compare species diversity, was applied to the data to estimate lipid diversity (cf., Meador et al., 2014). The *D* values range from 0 to 1, such that a value approaching 1 represents high lipid diversity and values close to 0 represent no diversity.

Principal component analysis (PCA) was performed on the raw MS chromatograms with Bruker Profile Analysis 2.0 in a *m/z* range from 600 to 2000 and between 10 and 25 min., thus including all lipids. Rectangular bucketing was established with dimensions of 1 min and 1 *m/z* units and data were normalized to the sum of buckets in each analysis. Samples were grouped according to treatments. In order to maximize comparability among samples for PCA, one replicate sample from hydrogen-limited experiments that was measured 2 weeks later than the others was excluded. Since main differences are expected to occur during the stationary phase, we also chose to exclude all L-Exp samples and include end-harvest samples of control and hydrogen-limited treatments. Because the latter are comparable to the L-Stat samples, they were not shown in IPL characterization.

## RESULTS AND DISCUSSION

### LIPID COMPOSITION OF *M. thermautotrophicus*

**FIGURE 1:**
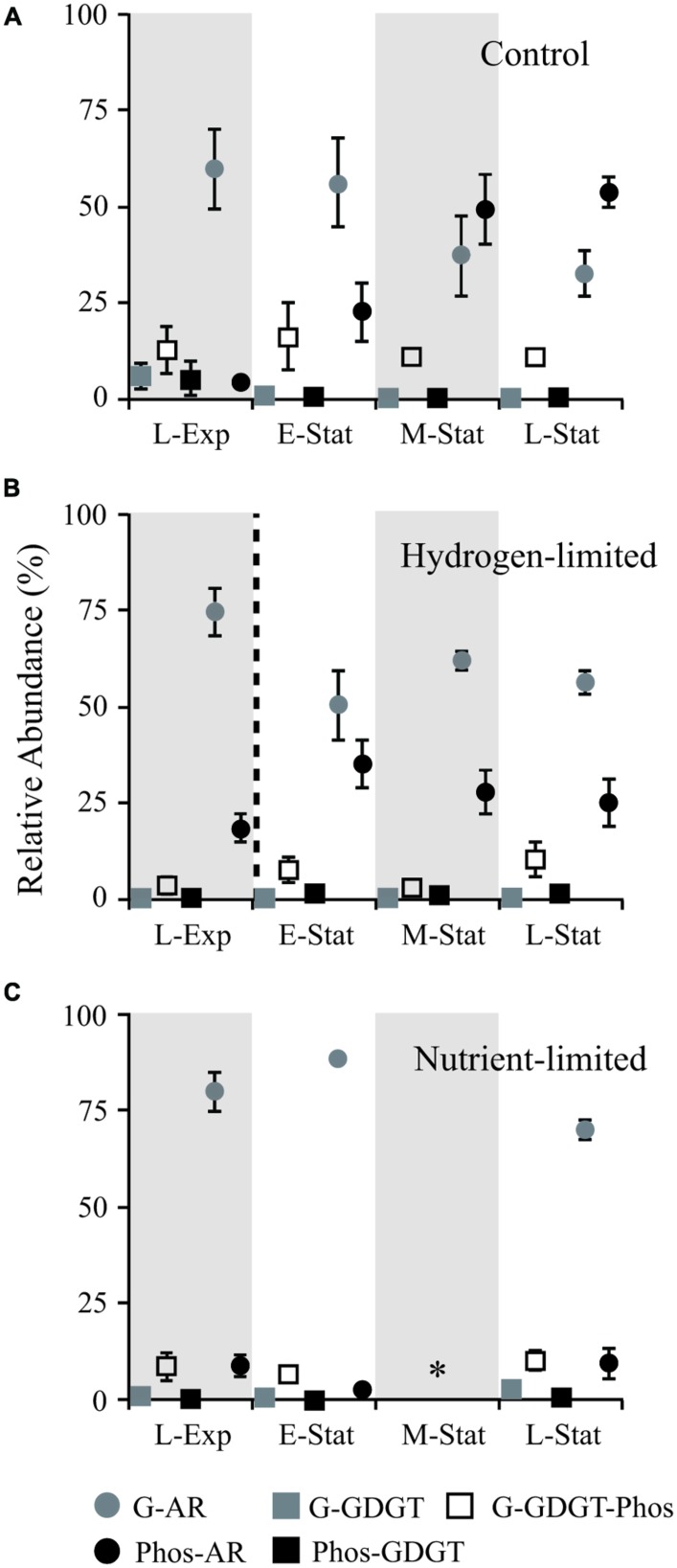
**Abundance of IPL relative to total lipids (including both core and IPL) during growth of *Methanothermobacter thermautotrophicus* under control **(A)**, hydrogen limiting **(B)**, and nutrient limiting **(C)** conditions at: late exponential phase (L-Exp), early, mid and late stationary phases (E-, M-, and L-Stat).** The dashed line in **(B)** denotes the change in headspace from 80:20 H_2_:CO_2_ to N_2_:CO_2_ (vol:vol) 3 h before sampling E-Stat in hydrogen-limited experiments. Star symbol (*) in **(C)** represents no data. The experiments were conducted in triplicate and error bars represent standard error of the mean. G, glycosidic; Phos, phosphatidic; AR, archaeols/diethers; GDGT, tetraethers.

**FIGURE 2:**
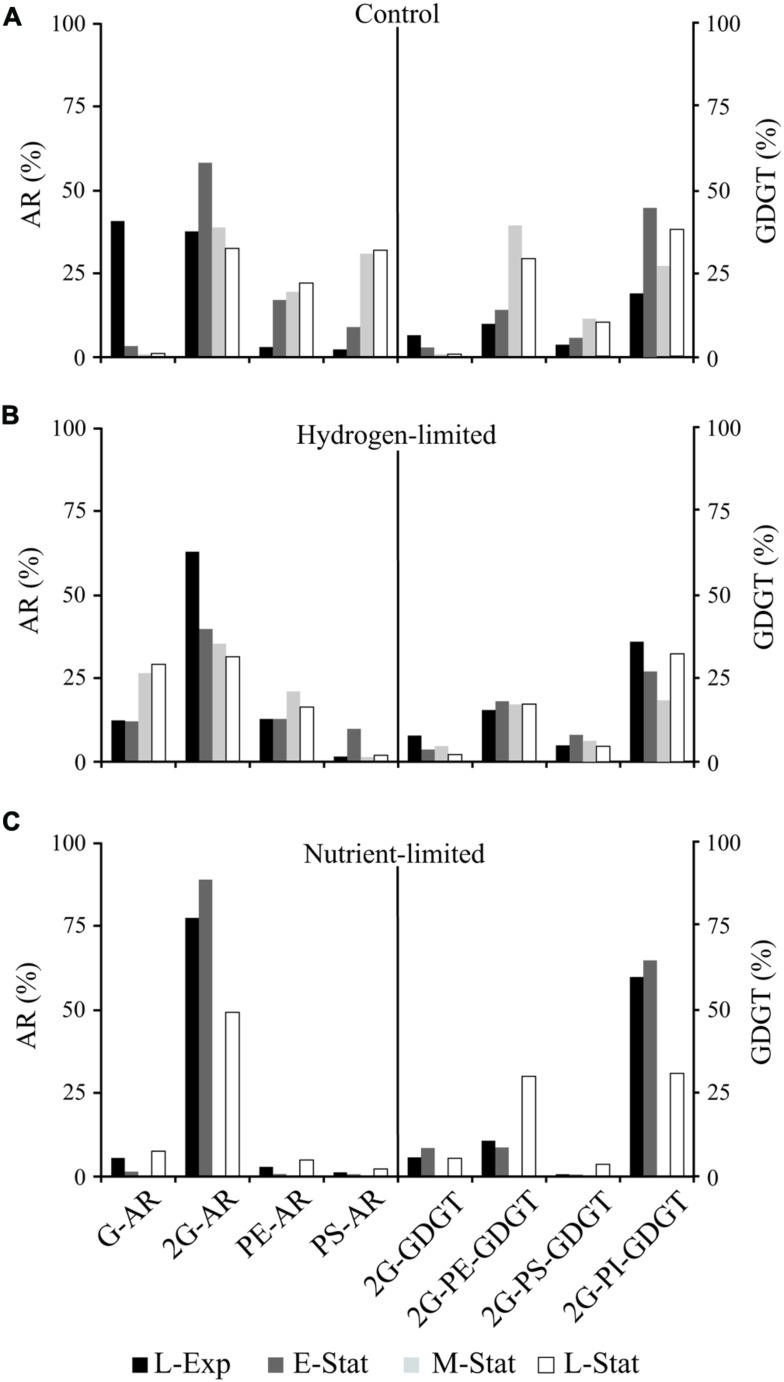
**Abundance of major individual AR and GDGT lipids relative to total diethers or tetraethers, respectively, from cells of *M. thermautotrophicus* growing under control **(A)**, hydrogen limiting **(B)**, and nutrient limiting **(C)** conditions.** For abbreviations see Supplementary Table S1.

Early investigations by thin layer chromatography coupled to fast atom bombardment MS or NMR have confirmed the identity of 23 different IPL in *M. thermautotrophicus* (Kramer et al., 1987; Nishihara et al., 1987, 1989). Based on HPLC-MS, our data show that *M. thermautotrophicus* ΔH (DSM 1053) membrane structure consists of ca. 65 different IPL, including diethers and tetraethers attached to glycosyl, phosphatidyl, and glycophosphatidyl headgroups (**Figure 1**). Nonetheless, with the exception of a relatively minor content of phosphatidyl-inositol (PI) AR, the major IPL reported in earlier studies (Nishihara et al., 1989; Morii and Koga, 1993) also represented dominant classes in the control samples in this study (**Figure 2A**).

Several IPL are reported here for the first time (Supplementary Tables S1 and S2; Supplementary Figure S1). To our knowledge, the C_15_–C_20_ archaeol (short-AR) has never been described, and this novel archaeal core lipid is exclusively bound to phosphatidyl-ethanolamine (PE) and phosphatidyl-serine (PS). The short-AR derivatives are likely synthesized via farnesyl diphosphate, since *M. thermautotrophicus* possesses genes encoding for short chain isoprenyl diphosphate synthase (Chen and Poulter, 1994). Although D-glucose has been reported as the sole sugar moiety in total lipid extracts from *M. thermautotrophicus* previously (Nishihara et al., 1987), the deoxy-glycosidic headgroup identified in our study appeared both in C_20_–C_20_ archaeol (AR) and glycerol-di-biphytanyl-glycerol-tetraethers (GDGT). Moreover, while sugar headgroups in *M. thermautotrophicus* have been reported exclusively as diglycosyl moieties (Morii et al., 2007), we also found the monoglycosyl derivatives of AR and GDGT. Novel glycosidic headgroups of AR were also detected, and included the tentatively identified phosphatidyl-*N*-acetylglycosaminyl, acetyl-, and/or glycerol–glycosyl moieties (Supplementary Figure S1).

While GDGT have been previously detected in *M. thermautotrophicus* with additional methylation in dibiphytanyl moieties (Knappy et al., 2009), our study observed for the first time that these methylated GDGT, with up to three methylations, were prevalent over acyclic C_40_–C_40_ GDGT (caldarchaeol) not only as core lipids, but also as IPL (Supplementary Table S1). Formation of tetraethers in *M. thermautotrophicus* is hypothesized to occur via head-to-head condensation of two diether lipids (e.g., Nishihara et al., 1989; Morii and Koga, 1994). Consistent with this model, the detection of a novel intact methylated C_21_–C_20_ AR (Supplementary Table S1, Supplementary Figure S1) suggests that the addition of a methyl group may occur in phytanyl chains, i.e., prior to the head-to-head condensation. Methylated GDGT seem to be a typical membrane lipid feature among thermophilic archaea, as they have been detected in both culture (*Methanopyrus kandleri*, Liu et al., 2012; *Thermococcus kodakarensis*, Meador et al., 2014) and environmental samples (Reeves et al., 2014).

### LIPID COMPOSITION IN RELATION TO GROWTH PHASE – CONTROL EXPERIMENTS

A higher proportion of diethers relative to tetraethers (87 ± 5%, *n* = 33 samples) was observed in all treatments and throughout the cell growth phases of *M. thermautotrophicus* (**Figure 1**). These results contrast with the drastic shifts either to higher proportion of diethers (Kramer and Sauer, 1991) or tetraethers (Morii and Koga, 1993) during the stationary phase of *M. thermautotrophicus*. The most likely reason for this noticeable discrepancy is that the previous studies employed distinct methods other than HPLC-MS for quantification of the diether/tetraether ratios (Iatroscan, which utilizes a flame ionization detector and ether cleavage and analysis of hydrocarbon chain by gas liquid chromatography, respectively). Nevertheless, in contrast to earlier reports of substantial shifts in core lipid composition but relatively constant headgroup composition throughout the growth phases, we observed striking differences in headgroup composition of both diether and tetraether IPL between different growth phases from *M. thermautotrophicus* control samples.

The most prominent feature observed within the control samples was the drastic decrease in proportions of glycosidic AR relative to total IPL during the transition from exponential growth to late stationary phases (**Figure 1A**). This change in IPL composition was associated with higher contributions of PS- and PE-AR to total diether lipids (**Figure 2A**), including minor AR attached to PI and phosphatidic acid (PA) headgroups. Although similar contributions of GDGT to total IPL were observed throughout the growth phases, our results revealed an increase in glycophosphatidyl headgroups, mainly G-PE and G-PI, and to a lesser extent G-PS. Reduced contributions of glycosidic AR toward the late-stationary phase were accompanied by relatively lower proportions of G-GDGT and headgroup-free core lipids (Supplementary Table S1).

This systematic substitution of glycosyl by phosphatidyl headgroups in diethers and tetraethers IPL classes is consistent with the putative hypothesis of GDGT biosynthesis via head-to-head condensation in archaea (e.g., *M. thermautotrophicus*, Nishihara et al., 1989; *T. acidophilum*, Nemoto et al., 2003). That is, an increase in phosphate-based AR was reflected in higher proportions of glycophosphatidyl GDGT in stationary growth phases (**Figure 2A**). The factors controlling IPL modification during growth phases under optimum conditions are still unknown, but could be related to the IPL orientation in the membrane bilayer (Morii and Koga, 1994) as will be discussed below in concert with low energy/nutrient treatments.

### LIPID COMPOSITION IN RESPONSE TO GROWTH-LIMITING CONDITIONS

In order to investigate the membrane lipid composition responses of *M. thermautotrophicus* to growth-limiting effects, we first examined the conditions under which growth was suppressed but not stopped (**Table 1**). Although slightly lower cell concentrations were observed during the transition from E-Stat to L-Stat in hydrogen limitation treatments, they were in the same order of magnitude compared to control. Maximum cell concentrations for nutrient-limited experiments were 20-times lower than those reached under control conditions, reflecting poor culture growth imposed by phosphate- and potassium-limiting conditions.

The drastic exchange of glycosyl by phosphatidyl AR observed during growth phases in control experiments was not observed in hydrogen- and nutrient-limited treatments (**Figures 1** and **2**). Rather, the gradual increase in phosphatidyl AR from L-Exp to E-Stat in hydrogen limitation experiments was interrupted soon after the gas exchange from H_2_:CO_2_ to N_2_:CO_2_, leading to a rapid decrease in the contribution of these lipids to the total IPL pool (**Figure 1B**). In nutrient limitation experiments, glycosidic AR represented the majority of IPL in *M. thermautotrophicus* and no major changes were observed throughout the growth stages.

Differences among treatments were also evidenced by the Simpson diversity index (*D*) and carbon-to-lipid ratio. While similar *D* values were obtained for control and hydrogen-limited treatments, the diversity of lipids was comparatively higher in nutrient limitation experiments regardless of the growth phase (Supplementary Figure S2). Cellular carbon-to-lipid ratios observed in control and hydrogen-limited treatments were highly variable (from 14 to 44), substantially higher than the values of 11 to 15 reported by Simon and Azam (1989) for planktonic bacteria. These ratios were relatively higher in nutrient-limited treatments, especially when reaching the E-Stat phase (56–81, Supplementary Figure S2).

**FIGURE 3:**
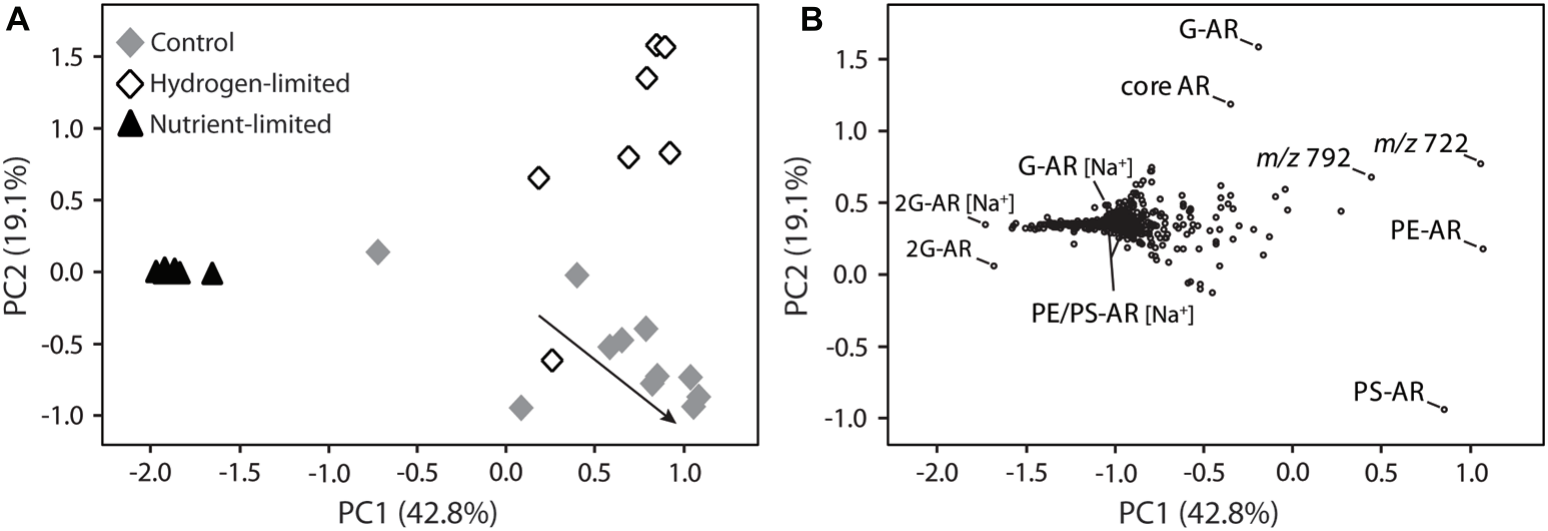
**Principal component analysis (PCA) of *M. thermautotrophicus* growing under control, hydrogen-limited and nutrient-limited conditions.** Bi-plots of two main principal components, PC1 and PC2, displayed as **(A)** samples from distinct treatments and **(B)** lipid distribution. The arrow in **(A)** illustrates control samples transitioning from late exponential growth to stationary phase. [Na^+^] indicates IPL bound to sodium and masses *m/z* 722 and 792 represent the most abundant unmodified polyprenols **(B)**, respectively, containing 10 and 11 isoprene units (see Supplementary Figure S4). For compounds abbreviations and detailed information on the PCA analysis please refer to Supplementary Table S1 and Section “Materials and Methods,” respectively.

To further assess IPL variability, changes in the relative abundance of individual compounds were evaluated by PCA, in which the first two principal components PC1 and PC2 explained 62% of the observed variance (**Figure 3**). Nutrient-limited samples clustered close to each other, displaying negative values in PC1 that were associated with 2G-AR with both ammonium and sodiated adducts. While low amounts of sodiated relative to ammonium adducts are produced during ESI ionization (e.g., Zhu et al., 2013), in this study we detected an extraordinarily high abundance of sodiated adducts in nutrient-limited samples (Supplementary Figure S3), suggesting that their occurrence reflects a biological response rather than an analytical phenomenon linked to the ionization conditions in the mass spectrometer. Because compelling evidence exists for the occurrence of 2G-AR naturally bound to Na^+^ and K^+^ in *M. thermautotrophicus* (Kramer et al., 1988), we investigated whether other forms of sodiated adducts were also present. Interestingly, sodiated forms of both glyco- and phospholipids, including the most abundant IPL (i.e., G-AR, PS-AR, and PE-AR), showed negative values in PC1 and did not cluster with the H^+^ or NH_4_^+^ counterparts (**Figure 3**), as would be expected if the different adducts were formed from the same compound during ESI ionization.

Control and hydrogen-limited samples generally displayed positive values in PC1, which were correlated with diagnostic IPL including the major diether phospholipids of *M. thermautotrophicus* (i.e., PE-AR and PS-AR) and two compounds with parent ions of *m/z* 792 and 722. Those latter compounds were identified as polyisoprenoidal alcohols or polyprenols, comprising 9–11 isoprene subunits (Supplementary Figure S4). While phosphate- or pyrophosphate-bound polyisoprenoids have been identified in eukaryotes, bacteria, and archaea (Swiezewska and Danikiewicz, 2005; Jones et al., 2009; Hartley and Imperiali, 2012), the polyprenols identified here did not possess phosphate (Supplementary Figure S4). Undecaprenyl monophosphate has been identified as the major sugar carrier for pseudomurein biosynthesis in *M. thermautotrophicus* (Hartmann and König, 1990). Although we cannot exclude a potential loss of monophosphate during TCA-based extraction (Lechner et al., 1985), phosphate-free polyprenols could play a distinct biochemical role in *M. thermautotrophicus* (e.g., membrane stability; Hartley and Imperiali, 2012).

Explaining 19% of the averaged IPL composition, PC2 also revealed trends differentiating the three treatments (**Figure 3**). The majority of samples from the hydrogen-limited experiments exhibited positive values in PC2, which were associated with monoglycosidic (G-AR) and core AR. Nutrient-limited samples clustered near to the origin, while most samples from control experiments clustered with negative values in PC2, which were strongly related to PS-AR.

### POSSIBLE MECHANISMS INVOLVED IN IPL MODIFICATION DURING GROWTH STAGES AND HYDROGEN/NUTRIENT LIMITATION

Morii and Koga (1994) found that the arrangement of IPL in the cell membrane of *M. thermautotrophicus* was asymmetrical and that most diglycosyl headgroups of both AR and GDGT as well as phosphoethanolamine bound to AR were located in the outer surface of the cell membrane. In contrast, the other phosphatidyl headgroups were found to face the cell interior, including serine and inositol headgroups of both AR and GDGT, and the ethanolamine from glycophosphatidyl GDGT. In our study, modifications in IPL were mostly related to the headgroup composition as similar ratios of tetraether relative to diether lipids were detected throughout the growth phases and among treatments. Therefore, positioning of IPL headgroups on the cell membrane of *M. thermautotrophicus* may be a critical response to energy and nutrient stress.

Shifts in IPL composition during growth stages in control experiments were larger than those observed in nutrient limitation treatments (**Figures 1** and **2**). This membrane lipid modification consisted of an increase in proportions of phosphatidyl relative to glycosyl headgroups of IPL toward the L-Stat, quite distinguishable from equivalent hydrogen-limited cells. One explanation for the accumulation of phosphatidyl IPL during the stationary phase is that these types of lipids have higher rates of lateral diffusion, i.e., higher permeability, than membranes made of glycolipids (Baba et al., 2001). The presence of more laterally mobile membranes could enhance rates of energy production at the expense of energy conservation (Valentine, 2007). *M. thermautotrophicus* may thus increase the proportion of glycolipids under energy and nutrient stress, decreasing the proton permeability of cell membranes.

A high proportion of glyco-relative to phospholipids was indeed observed in growth-limiting treatments. However, whereas 2G-AR represented the majority of IPL in nutrient-limited experiments, increased abundances of G-AR and G-GDGT were observed toward the L-Stat in hydrogen-limited conditions (**Figure 2**; Supplementary Table S1). As opposed to bilayer-forming diglycosidic headgroups, the substitution of phospho- by monoglycosidic headgroups under hydrogen limitation might be linked to their non-bilayer properties and associated lipid-protein interactions at the membrane level, similarly to observations in bacterial (Wikström et al., 2004) and photosynthetic cells (Hölzl and Dörmann, 2007). On the other hand, the reason for a relatively larger quantity of diglycosidic IPL in nutrient limitation treatments may be related to the formation of hydrogen-bond network among sugar headgroups (Hinz et al., 1991; Baba et al., 2001), similarly to *T. acidophilum* in response to low pH and high temperature (Shimada et al., 2008). By increasing the number of glycosyl headgroups per unit of IPL, *M. thermautotrophicus* may decrease membrane fluidity and protect cell membranes, particularly at the outer face, from chemically unstable conditions.

In comparison with the other treatments, *M. thermautotrophicus* cells under nutrient limitation were also characterized by a relatively lower importance of polyprenols and by the synthesis of IPL tightly bound to Na^+^ (**Figure 3** and Supplementary Figure S3). Under potassium limiting conditions, *M. thermautotrophicus* has been shown to accumulate intracellular K^+^, generating gradients as large as 50,000-fold between internal and external concentrations (Schönheit et al., 1984; Krueger et al., 1986). Rather than being driven by membrane potential or external osmotic pressure, this phenomenon could be related to the accumulation of the cyclic 2,3-diphosphoglycerate (cDPG), which bears a -3 charge at physiological pH and could function as a counter ion for K^+^ accumulation (Krueger et al., 1986). cDPG represents a major carbon- and phosphorous-containing solute in *M. thermautotrophicus* (Seely and Fahrney, 1983; Evans et al., 1985) and due to its accumulation, phosphorous-limited cells exhibit a total phosphorus content comparable to that of cells from optimized batch cultures (Seely and Fahrney, 1984; Krueger et al., 1986). Since salt forms of lipids in *Methanosarcina mazei* have been demonstrated to add stability to the cells by influencing the permeability of membranes to retain low molecular weight compounds (Patel et al., 1999), it is conceivable to propose that IPL bound to Na^+^ in *M. thermautotrophicus* are used to maintain essential intracellular concentrations of cDPG under nutrient-limited conditions. The accumulation of cDPG would also explain the high carbon-to-lipid ratios observed in early growth stages of these cells (Supplementary Figure S2). The substantially lower cell concentrations in nutrient-limited experiments compared to the other treatments could have been triggered by the lack of available phosphorus as an essential cellular component. Moreover, these lower cell concentrations could possibly reflect the increased energy demand on cells related to K^+^ uptake and maintenance of K^+^ concentration gradients (Schönheit et al., 1984; Krueger et al., 1986). The unique lipid profile exhibited by nutrient-limited cells can thus be considered a response of *M. thermautotrophicus* to chronic stress.

Although displaying similar cell concentrations, carbon-to-lipid ratios and IPL diversity in comparison with control experiments, cells growing under hydrogen stress presented very distinct IPL composition as evidenced by PCA analysis, including an apparently high abundance of polyprenols. With the exception of nutrient limitation treatments, polyprenols were extremely abundant in all our samples, accounting for up to 40% of total lipids (Supplementary Table S1). While the function of phosphate-free polyprenols is still unclear, there exists significant evidence for an increase in fluidity and ion permeability of membranes, including changes in membrane conformation (e.g., Hartley and Imperiali, 2012). Under hydrogen limitation, it has been reported that *M. thermautotrophicus* increases its cell wall thickness (Nakamura et al., 2006) and upregulates genes related to polysaccharide biosynthesis (Kato et al., 2008). Given the importance of their phosphate-bearing counterparts as sugar carriers for pseudomureins synthesis in *M. thermautotrophicus* (Hartmann and König, 1990), the high abundance of polyprenols may alternatively reflect a link with the production of pseudomureins (Kandler and König, 1998), for the purpose of cell wall thickening in response to energy stress.

## CONCLUSION

Although archaeal lipids have been studied for many years, innovative HPLC-MS methods have allowed the characterization of novel molecular structures in a single analytical run, which are useful to investigate detailed membrane lipid composition of archaea both in cultures and natural systems. In* M. thermautotrophicus*, methylated forms of GDGT predominate over caldarchaeol. Additionally, methylation of caldarchaeol represents a characteristic feature among thermophilic archaea and has implications for studying thermophilic lipids in environmental samples (e.g., hydrothermal systems) and in biotechnology (e.g., permeability of liposomes).

*Methanobacterium thermautotrophicus* responded similarly to growth under energy and nutrient limitation. That is, cell membranes under these growth-limiting conditions were dominated by glycolipids, which face the outer leaflet of the membrane bilayer, as opposed to the higher abundance of phospholipids in control experiments. While this major lipid shift could be attributed to regulatory mechanisms of cell membrane permeability in *M. thermautotrophicus*, the role of membrane lipids is much more diverse as they actively participate in various essential cellular processes. A headgroup substitution in IPL from growth-limited cells may implicate regulation of lipid-protein interactions, causing protein conformation changes, with crucial consequences for the balance between energy production and conservation.

In agreement with previously observed increases in cell wall thickness for energy-limited *M. thermautotrophicus*, specific lipid modifications under hydrogen depletion included the accumulation of polyprenols, which may play a role in the synthesis of pseudomureins. If connected to the increased proportion of glycolipids that provide a more effective barrier to ions, the reason for increasing cell wall thickness might be cellular regulation to decrease membrane permeability and leakage of ions in response to energy limiting conditions. These observations are in line with the varying H^+^/ATP or Na^+^/ATP stoichiometry, i.e., the protons translocated per ATP formed by *M. thermautotrophicus* according to the availability of energy (Poorter et al., 2003).

Despite exhibiting low contents of polyprenols and substantially lower cell concentrations in comparison with other treatments, *M. thermautotrophicus* cells under potassium and phosphate limitation were characterized by a surprisingly high content of salt forms of lipids. While the function of these lipids bound to Na^+^ in *M. thermautotrophicus* is still uncertain, they may be related to the retention of cDPG, which is essential to counterbalance the intracellular accumulation of K^+^ under potassium limiting conditions. Collectively our findings suggest that *M. thermautotrophicus* intensely modulates its cell membrane lipid composition to cope with energy and nutrient availability in dynamic environments.

## Conflict of Interest Statement

The authors declare that the research was conducted in the absence of any commercial or financial relationships that could be construed as a potential conflict of interest.
