# Supplementary material for: Methanothermobacter thermautotrophicus modulates its membrane lipids in response to hydrogen and nutrient availability
Source: Front Microbiol. 2015 Jan 22;6:5. doi: 10.3389/fmicb.2015.00005 (PMC4302986; doi:10.3389/fmicb.2015.00005)
Supplement: Supplementary file 1 [file Data_Sheet_1.DOCX]

***Supplementary Material***

***Methanothermobacter thermautotrophicus* modulates its membrane lipids in response to hydrogen and nutrient availability**

**Marcos Y. Yoshinaga^1,a,*^, Emma J. Gagen^2,a,†^, Lars Wörmer^1,a^, Nadine K. Broda^1^, Travis B. Meador^1^, Jenny Wendt^1^, Michael Thomm^2^, Kai-Uwe Hinrichs^1^**

^1^MARUM Center for Marine Environmental Sciences, University of Bremen, Germany.

^2^Department of Microbiology and Archaea Center, University of Regensburg, Germany.

^a^These authors contributed equally to the manuscript

†present address: School of Earth Sciences, The University of Queensland, St Lucia, Australia.

*** Correspondence:** Marcos Yukio Yoshinaga, MARUM Center for Marine Environmental Sciences – Leobener Straβe, Bremen 28359, GERMANY – Tel.: +49 421 218 65744.

marcosyukio@gmail.com

1. **Supplementary Figures and Tables**

## Suplementary Tables

**Supplementary Table 1. Lipids of *M. thermautotrophicus* during growth stages for control, hydrogen- and nutrient-limited experiments: late exponential (L-Exp), early, mid and late stationary phase (E-, M- and L-Stat). *Lipids in bold represent either new occurrence for *M. thermautotrophicus* or novel archaeal lipids. Concentrations of individual lipids are given in µg g^-1^ of dry mass. The relative abundance of polyprenols (%) and total organic carbon content (%) are also reported.**

|  | **Control** | | | |  | **Hydrogen Limiting** | | | |  | **Nutrient Limiting** | | |
| --- | --- | --- | --- | --- | --- | --- | --- | --- | --- | --- | --- | --- | --- |
| **Lipid*** | **L-Exp** | **E-Stat** | **M-Stat** | **L-Stat** |  | **L-Exp** | **E-Stat** | **M-Stat** | **L-Stat** |  | **L-Exp** | **E-Stat** | **L-Stat** |
| **Diethers (AR)** |  |  |  |  |  |  |  |  |  |  |  |  |  |
| **core AR** | 1166 | 61 | 65 | 61 |  | 480 | 584 | 841 | 1416 |  | 131 | 51 | 65 |
| **G-AR** | 5393 | 151 | 108 | 114 |  | 3604 | 1908 | 4501 | 6467 |  | 322 | 52 | 100 |
| **deoxyG-AR** | 1040 | 314 | 309 | 292 |  | 730 | 421 | 614 | 902 |  | 164 | 108 | 256 |
| **2G-AR** | 7748 | 3534 | 6714 | 3688 |  | 22452 | 5613 | 5341 | 5602 |  | 4555 | 3057 | 2432 |
| **GAcG-AR** | 70 | 24 | 19 | 16 |  | 62 | 23 | 14 | 25 |  | 19 | 16 | 33 |
| **gGG-AR** | 3 | 9 | 7 | 9 |  | 1 | 0 | 0 | 1 |  | 10 | 3 | 3 |
| **gGGAc-AR** | 8 | 16 | 10 | 9 |  | 6 | 1 | 3 | 2 |  | 17 | 8 | 7 |
| **PGNAc-AR (uns)** | 14 | 47 | 124 | 77 |  | 180 | 116 | 26 | 48 |  | 135 | 44 | 71 |
| **PI-AR** | 67 | 60 | 424 | 389 |  | 514 | 433 | 343 | 1157 |  | 218 | 16 | 62 |
| **PS-AR** | 393 | 592 | 5426 | 3735 |  | 343 | 788 | 173 | 335 |  | 98 | 21 | 108 |
| **PS-AR (+CH_2_)** | 58 | 39 | 202 | 80 |  | 1350 | 594 | 580 | 663 |  | 111 | 35 | 49 |
| **PS-AR (uns)** | 0 | 6 | 79 | 47 |  | 3 | 12 | 2 | 4 |  | 1 | 0 | 5 |
| **PS-short-AR** | 0 | 4 | 71 | 56 |  | 1 | 7 | 2 | 4 |  | 0 | 0 | 6 |
| **PE-AR** | 408 | 978 | 3221 | 2542 |  | 3299 | 2854 | 3218 | 2842 |  | 223 | 21 | 283 |
| **PE-AR (uns)** | 0 | 17 | 82 | 62 |  | 57 | 67 | 60 | 57 |  | 7 | 4 | 52 |
| **PE-short-AR** | 0 | 10 | 50 | 41 |  | 23 | 34 | 27 | 31 |  | 4 | 3 | 35 |
| **PA-AR** | 38 | 95 | 332 | 322 |  | 77 | 88 | 29 | 79 |  | 28 | 11 | 6 |
| **Tetraethers (GDGT)** |  |  |  |  |  |  |  |  |  |  |  |  |  |
| **core GDGT** | 942 | 29 | 18 | 9 |  | 35 | 13 | 10 | 11 |  | 13 | 6 | 789 |
| **core (+CH_2_)** | 166 | 2 | 1 | 1 |  | 14 | 10 | 7 | 11 |  | 14 | 6 | 6 |
| **core (+C_2_H_4_)** | 35 | 1 | 0 | 0 |  | 2 | 2 | 1 | 2 |  | 2 | 1 | 1 |
| **G** | 570 | 15 | 7 | 4 |  | 26 | 16 | 17 | 18 |  | 8 | 3 | 4 |
| **G (+CH_2_)** | 336 | 5 | 2 | 2 |  | 20 | 18 | 26 | 30 |  | 11 | 5 | 6 |
| **G (+C_2_H_4_)** | 83 | 2 | 1 | 1 |  | 2 | 3 | 4 | 5 |  | 1 | 1 | 1 |
| **G (uns +CH_2_)** | 66 | 1 | 0 | 0 |  | 0 | 4 | 5 | 8 |  | 2 | 1 | 1 |
| **G (uns +C_2_H_4_)** | 22 | 0 | 0 | 0 |  | 7 | 5 | 7 | 7 |  | 2 | 0 | 0 |
| **deoxyG (+CH_2_)** | 0 | 0 | 0 | 0 |  | 0 | 0 | 0 | 0 |  | 0 | 0 | 0 |
| **deoxyG (+C_2_H_4_)** | 45 | 1 | 0 | 0 |  | 1 | 2 | 2 | 4 |  | 1 | 0 | 0 |
| **2G** | 268 | 8 | 5 | 4 |  | 46 | 13 | 20 | 20 |  | 18 | 9 | 11 |
| **2G (+CH_2_)** | 590 | 9 | 6 | 6 |  | 36 | 16 | 23 | 24 |  | 25 | 14 | 16 |
| **2G (+C_2_H_4_)** | 251 | 3 | 2 | 1 |  | 3 | 2 | 2 | 3 |  | 3 | 3 | 2 |
| **2G (+C_3_H_6_)** | 0 | 0 | 0 | 0 |  | 0 | 0 | 0 | 0 |  | 0 | 0 | 0 |
| **deoxy3G** | 0 | 1 | 2 | 1 |  | 7 | 2 | 1 | 1 |  | 1 | 0 | 0 |
| **deoxy3G (+CH_2_)** | 1 | 2 | 4 | 2 |  | 9 | 3 | 2 | 2 |  | 3 | 2 | 2 |
| **4G** | 4 | 4 | 5 | 4 |  | 9 | 6 | 1 | 10 |  | 4 | 1 | 2 |
| **4G (+CH_2_)** | 0 | 2 | 3 | 2 |  | 4 | 3 | 1 | 6 |  | 5 | 3 | 3 |
| **G PE** | 127 | 4 | 5 | 4 |  | 22 | 31 | 45 | 71 |  | 2 | 1 | 2 |
| **G PE (+CH_2_)** | 196 | 7 | 10 | 6 |  | 13 | 48 | 74 | 134 |  | 2 | 1 | 4 |
| **G PE (+C_2_H_4_)** | 350 | 5 | 3 | 1 |  | 7 | 9 | 9 | 20 |  | 2 | 0 | 2 |
| **deoxyG PE** | 20 | 17 | 18 | 12 |  | 10 | 9 | 9 | 9 |  | 1 | 0 | 1 |
| **deoxyG PE (+CH_2_)** | 182 | 13 | 32 | 20 |  | 9 | 13 | 12 | 18 |  | 1 | 1 | 6 |
| **deoxyG PE (+C_2_H_4_)** | 18 | 0 | 11 | 5 |  | 0 | 4 | 6 | 6 |  | 0 | 0 | 4 |
| **2G PE** | 272 | 53 | 254 | 139 |  | 72 | 88 | 43 | 169 |  | 20 | 4 | 18 |
| **2G PE (+CH_2_)** | 728 | 100 | 533 | 282 |  | 108 | 184 | 88 | 377 |  | 63 | 17 | 111 |
| **2G PE (+C_2_H_4_)** | 50 | 10 | 74 | 34 |  | 8 | 17 | 7 | 32 |  | 8 | 7 | 50 |
| **2G PS** | 90 | 16 | 87 | 62 |  | 18 | 19 | 4 | 27 |  | 2 | 0 | 1 |
| **2G PS (+CH_2_)** | 218 | 41 | 181 | 110 |  | 34 | 71 | 22 | 89 |  | 2 | 0 | 7 |
| **2G PS (+C_2_H_4_)** | 0 | 7 | 31 | 23 |  | 7 | 15 | 5 | 20 |  | 0 | 0 | 4 |
| **2G PA** | 108 | 7 | 14 | 11 |  | 28 | 17 | 11 | 115 |  | 11 | 1 | 0 |
| **2G PA (+CH_2_)** | 96 | 10 | 24 | 21 |  | 31 | 46 | 40 | 431 |  | 21 | 3 | 1 |
| **PI** | 114 | 9 | 29 | 18 |  | 38 | 62 | 24 | 104 |  | 12 | 3 | 7 |
| **PI (+CH_2_)** | 414 | 13 | 45 | 25 |  | 25 | 101 | 52 | 276 |  | 15 | 5 | 9 |
| **PI (+C_2_H_4_)** | 196 | 5 | 7 | 5 |  | 13 | 23 | 13 | 60 |  | 2 | 1 | 3 |
| **deoxyG PI** | 0 | 3 | 22 | 13 |  | 1 | 4 | 3 | 16 |  | 1 | 0 | 0 |
| **deoxyG PI (+CH_2_)** | 47 | 18 | 81 | 47 |  | 3 | 19 | 8 | 46 |  | 0 | 0 | 4 |
| **deoxyG PI (+C_2_H_4_)** | 7 | 3 | 20 | 9 |  | 0 | 4 | 1 | 8 |  | 1 | 1 | 1 |
| **2G PI** | 353 | 122 | 131 | 147 |  | 192 | 69 | 47 | 306 |  | 70 | 16 | 23 |
| **2G PI (+CH_2_)** | 633 | 379 | 469 | 459 |  | 336 | 239 | 142 | 1018 |  | 289 | 122 | 183 |
| **2G PI (+C_2_H_4_)** | 73 | 50 | 80 | 78 |  | 22 | 28 | 18 | 130 |  | 58 | 64 | 103 |
| **2G PI (uns +CH_2_)** | 31 | 16 | 13 | 23 |  | 12 | 6 | 2 | 26 |  | 13 | 7 | 11 |
| **PI PE** | 0 | 4 | 15 | 17 |  | 1 | 0 | 0 | 0 |  | 0 | 0 | 0 |
| **PI PE (+CH_2_)** | 36 | 10 | 28 | 32 |  | 0 | 1 | 1 | 2 |  | 0 | 0 | 0 |
| **PI PE (+C_2_H_4_)** | 0 | 0 | 1 | 2 |  | 0 | 0 | 0 | 0 |  | 0 | 0 | 0 |
| **PE** | 193 | 8 | 8 | 6 |  | 19 | 52 | 48 | 50 |  | 3 | 0 | 2 |
| **PE (+CH_2_)** | 834 | 19 | 15 | 10 |  | 17 | 67 | 88 | 89 |  | 3 | 1 | 3 |
| **PE (+C_2_H_4_)** | 602 | 8 | 2 | 1 |  | 4 | 12 | 11 | 11 |  | 0 | 0 | 2 |
| **PE PE** | 156 | 4 | 13 | 9 |  | 6 | 1 | 0 | 1 |  | 0 | 0 | 0 |
| **PE PE (+CH_2_)** | 86 | 5 | 15 | 14 |  | 0 | 1 | 1 | 0 |  | 0 | 0 | 0 |
| **PE PE (+C_2_H_4_)** | 0 | 0 | 2 | 2 |  | 0 | 0 | 0 | 0 |  | 0 | 0 | 0 |
| **Sum**** | 26.0 | 7.0 | 19.6 | 13.2 |  | 34.5 | 14.9 | 16.7 | 23.5 |  | 6.8 | 3.8 | 4.2 |
| **Polyprenols (%)***** | 17 | 27 | 30 | 32 |  | 18 | 25 | 26 | 22 |  | 6 | 10 | 9 |
| **Glycosylated polyprenols (%)** | 14 | 8 | 5 | 4 |  | 7 | 6 | 15 | 19 |  | 7 | 15 | 27 |
| **%TOC (std. deviation)** | 29 (5) | 36 (1) | 33 (5) | 36 (1) |  | 34 (2) | 35 (1) | 37 (3) | 35 (1) |  | 14 (1) | 23 (4) | 20 (2) |
| ** the sum of lipids is given in mg g^-1^ of dry mass. *** percentage of polyprenols relative to total lipids (i.e. core and polar lipids plus polyprenols). Abbreviations: AR=diphytanyl archaeol, G=monoglycosyl, deoxyG=deoxyglycosyl, gG=glycerolglycosyl, GAc=acetylglycosyl, PGNAc=Phosphatidyl-N-acetylglycosaminyl, PE=phosphatidyl-ethanolamine, uns=monounsaturated, PI=phosphatidyl-inositol, PS=phosphatidyl-serine, +CH_2_=methylated, +C_2_H_4_=dimethylated, +C_3_H_6_=trimethylated, short-AR=C_15_-C_20_ AR, PA=phosphatidic acid, GDGT=glycerol-dibiphytanyl-glycerol-tetraethers. | | | | | | | | | | | | | |

**Supplementary Table 2. Exemplary fragmentation patterns of novel diethers (AR) and tetraethers (GDGT) of *M. thermautotrophicus* cells by HPLC-ESI-MS. For abbreviation see Supplementary Table 1.**

| IPL | Molecular  Formula | | | m/z | | I.C. | | Major  Fragment(s) | | Loss(es) |  |  |  |  |  |
| --- | --- | --- | --- | --- | --- | --- | --- | --- | --- | --- | --- | --- | --- | --- | --- |
| deoxyG-AR | C_49_H_102_NO_7_^+^ | | | 816.765 | | C_40_ | | 653.681 | | deoxyG + NH_3_^+^ |  |  |  |  |  |
|  |  |  |  |  |  |  |  | 373.368 | | deoxyG + C_20_ |  |  |  |  |  |
| GAcG-AR | C_57_H_114_NO_14_^+^ | | | 1036.823 | | C_40_ | | 653.681 | | GAcG + NH_3_^+^ |  |  |  |  |  |
|  |  |  |  |  |  |  |  | 373.368 | | GAcG + C_20_ |  |  |  |  |  |
| gGG-AR | C_58_H_118_NO_15_^+^ | | | 1068.85 | | C_40_ | | 653.681 | | gGG + NH_3_^+^ |  |  |  |  |  |
| gGGAc-AR | C_60_H_120_NO_16_^+^ | | | 1110.86 | | C_40_ | | 653.681 | | gGGAc + NH_3_^+^ |  |  |  |  |  |
|  |  |  |  |  |  |  |  | 373.368 | | gGGAc + C_20_ |  |  |  |  |  |
| PGNAc-AR | C_51_H_103_NO_11_P^+^ | | | 936.726 | | C_40_ | | 733.647 | | PGNAc |  |  |  |  |  |
|  |  |  |  |  |  |  |  | 204.087 | | PA-AR |  |  |  |  |  |
| PS-AR (+CH_2_) | C_47_H_97_NO_8_P^+^ | | | 834.695 | | C_41_ | | 747.663 | | Serine |  |  |  |  |  |
|  |  |  |  |  |  |  |  | 733.647 | | Serine + CH_2_ |  |  |  |  |  |
|  |  |  |  |  |  |  |  | 453.334 | | Serine + C_21_ |  |  |  |  |  |
| PS-AR (uns) | C_46_H_93_NO_8_P^+^ | | | 818.663 | | C_40:1_ | | 540.366 | | C_20:1_ |  |  |  |  |  |
|  |  |  |  |  |  |  |  | 453.334 | | Serine + C_20:1_ |  |  |  |  |  |
| PS-short-AR | C_41_H_85_NO_8_P^+^ | | | 750.601 | | C_35_ | | 663.569 | | Serine |  |  |  |  |  |
|  |  |  |  |  |  |  |  | 453.334 | | Serine + C_15_ |  |  |  |  |  |
| PE-AR (uns) | C_45_H_93_NO_6_P^+^ | | | 774.674 | | C_40:1_ | | 496.376 | | C_20:1_ |  |  |  |  |  |
|  |  |  |  |  |  |  |  | 453.334 | | Ethanolamine + C_20:1_ |  |  |  |  |  |
| PE-short-AR | C_40_H_85_NO_6_P^+^ | | | 706.611 | | C_35_ | | 663.569 | | Ethanolamine |  |  |  |  |  |
|  |  |  |  |  |  |  |  | 453.334 | | Ethanolamine + C_15_ |  |  |  |  |  |
|  |  | | |  | |  | |  | |  |  |  |  |  |  |
| G-GDGT | C_92_H_186_NO_11_^+^ | | | 1481.402 | | C_80_ | | 1302.323 | | G + NH_3_^+^ |  |  |  |  |  |
| G-GDGT (+CH_2_) | C_93_H_188_NO_11_^+^ | | | 1495.418 | | C_81_ | | 1316.338 | | G + NH_3_^+^ |  |  |  |  |  |
| deoxyG-GDGT | C_93_H_188_NO_10_^+^ | | | 1479.423 | | C_80_ | | 1316.338 | | deoxyG + NH_3_^+^ |  |  |  |  |  |
| 2G-GDGT-PI (+CH_2_) | C_105_H_209_NO_24_P^+^ | | | 1899.49 | | C_81_ | | 1882.463 | | NH_3_^+^ |  |  |  |  |  |
|  |  |  |  |  |  |  |  | 1720.41 | | G + NH_3_^+^ |  |  |  |  |  |
|  |  |  |  |  |  |  |  | 1558.357 | | 2G + NH_3_^+^ |  |  |  |  |  |
| 2G-GDGT-PI (+C_2_H_4_) | C_106_H_211_NO_24_P^+^ | | | 1913.505 | | C_82_ | | 1896.479 | | NH_3_^+^ |  |  |  |  |  |
|  |  |  |  |  |  |  |  | 1572.373 | | 2G + NH_3_^+^ |  |  |  |  |  |
| I.C.=isoprenoidal chain length | | | | | | | | | | |  |  |  |  |  |
|  | | | | | | | | | | |  |  |  |  |  |
|  | |  |  | |  | |  | |  | |  |  |  |  |  |

## Supplementary Figures

Supplementary Figure 1. Novel occurrences of diether lipids in *M. thermautotrophicus* and novel archaeal lipids tentatively identified by ESI-MS/MS.

Supplementary Figure 2. Lipids Simpson diversity index (a) and carbon to lipid ratios (b) of *M. thermautotrophicus* growing under control, hydrogen-limited and nutrient-limited conditions. Error bars represent standard error of the mean (n=3). For detailed information on these analyses please see to the Methods section.

**Supplementary Figure 3. Exemplary spectra of G-AR obtained by reverse-phase/positive-mode HPLC-ESI-MS in MS^1^ mode. The molecular masses at 832.7 and 837.7 illustrate respectively G-AR ionized with NH_4_^+^ and Na^+^ in control, hydrogen limiting and nutrient limiting conditions. Note that the intensity of G-AR ionized with Na^+^ is comparable to that with NH_4_^+^ only in the nutrient-limited sample. Confirmation of sodium ion association with IPL was performed by multiple measurements from the three treatments.**

**Supplementary Figure 4. Density map showing major diether lipids of *M. thermautotrophicus* and their elution time during reverse-phase/positive-mode HPLC-ESI-MS in MS^1^ mode. Zoomed area illustrates the presence of polyprenols containing 9 to 11 isoprene subunits and different degrees of unsaturation.**
